# Association Between Internet Use and Sleep Health Among Middle-Aged and Older Chinese Individuals: Nationwide Longitudinal Study

**DOI:** 10.2196/71030

**Published:** 2025-04-16

**Authors:** Xueqin Li, Jin Liu, Ning Huang, Wanyu Zhao, Hongbo He

**Affiliations:** 1 Guangdong Cardiovascular Institute Guangdong Provincial People's Hospital Ganzhou Hospital Guangdong Academy of Medical Sciences Guangzhou China; 2 Guangdong Mental Health Center Guangdong Provincial People's Hospital, Guangdong Academy of Medical Sciences Southern Medical University Guangzhou China; 3 National Clinical Research Center for Mental Disorders Department of Psychiatry The Second Xiangya Hospital of Central South University Changsha China; 4 Mental Health Institute of Central South University, China National Clinical Research Center on Mental Disorders (Xiangya) China National Technology Institute on Mental Disorders, Hunan Technology Institute of Psychiatry Hunan Key Laboratory of Psychiatry and Mental Health Changsha China; 5 Center of Gerontology and Geriatrics and National Clinical Research Center of Geriatrics West China Hospital Sichuan University Chengdu China

**Keywords:** internet use, sleep, Chinese middle-aged and older adults, internet frequency, cohort study

## Abstract

**Background:**

Sleep disorders are common among older adults and have a bidirectional impact on their emotional well-being. While some studies suggest that internet use may offer mental health benefits to this population, the relationship between internet use and sleep outcomes remains underexplored.

**Objective:**

This study investigates the association between internet use (including use frequency) and sleep quality and duration in middle-aged and older Chinese adults.

**Methods:**

A longitudinal analysis was conducted using the China Health and Retirement Longitudinal Study data from 2015 to 2018. Sleep quality was assessed using the sleep item from the Centre for Epidemiologic Studies Depression Scale, categorized as “good” (<1 day; reference), “fair” (1-4 days), or “poor” (5-7 days). Sleep duration was classified as short (<6 hours), medium (6-9 hours; reference), or long (>9 hours). Adjusted multinomial logistic regressions were used to examine the associations between internet use or frequency in 2015 and sleep quality or duration in 2018, controlling for age, sex, residence, diseases, smoking, drinking, and napping time and further exploring sex and age group variations.

**Results:**

The baseline analysis included 18,460 participants aged 45 years and older, with 1272 (6.9%) internet users, 8825 (48.1%) participants had fair or poor sleep, and 6750 (37.2%) participants had abnormal sleep duration. Internet users, particularly those who used it almost daily, were less likely to report poor sleep quality (relative risk [RR] 0.71, 95% CI 0.54-0.94) and longer sleep duration (RR 0.22, 95% CI 0.11-0.44) than nonusers. In the longitudinal analysis, baseline internet users had a significantly reduced risk of fair (RR 0.66, 95% CI 0.51-0.86) and poor sleep quality (RR 0.60, 95% CI 0.44-0.81), as well as short (RR 0.73, 95% CI 0.53-1.00) and long sleep duration (RR 0.39, 95% CI 0.21-0.72) during the follow-up period than nonusers. These associations remained significant for almost daily internet use (RR 0.32, 95% CI 0.15-0.69). Subgroup analyses by sex revealed a positive relationship between internet use and sleep quality, with a stronger effect in female (poor sleep: RR 0.57, 95% CI 0.36-0.89) than male (poor sleep: RR 0.61, 95% CI 0.40-0.92) participants. The effect on sleep duration was significant only in daily male users, showing a reduced risk of long sleep duration (RR 0.30, 95% CI 0.11-0.78). In the age subgroup analysis, most internet users were in the 45- to 59-year age group, with results consistent with the overall findings.

**Conclusions:**

This study suggests that internet use is associated with a reduced risk of sleep problems in middle-aged and older adults. The findings indicate that moderate, regular internet engagement—such as daily use—may promote better sleep health in this population.

## Introduction

Human sleep patterns change with age, and older adults experience systematic declines in sleep efficiency [1,2]. The aging population is progressing rapidly. By the mid-2030s, the population of individuals aged 80 years and older will reach 265 million, surpassing the number of infants [3]. Additionally, China’s Ministry of Civil Affairs reports that by the end of 2023, the population of individuals aged 60 years and older is approaching 297 million, making up 21.1% of the total population [4]. Consequently, the number of older adults experiencing sleep problems is also rising at a significant rate. One in 2 older adults reports experiencing difficulty sleeping [5]. Sleep problems, including insufficient or excessive sleep durations and poor sleep quality, are highly prevalent among older adults and have been linked to adverse health outcomes, including premature mortality, cognitive decline, cardiovascular disease, diabetes, Alzheimer dementia, and functional impairment [6-13]. Therefore, exploring effective interventions to improve sleep in older adults holds significant public health importance. Although pharmacological interventions are commonly used to manage sleep disorders, long-term use often poses risks such as falls and cognitive impairment in older adults [14,15], underscoring the need for practical nonpharmacological approaches to address sleep issues in middle-aged and older populations. Existing studies have shown that exercise interventions [16,17], cognitive behavioral therapy for insomnia [18-20], and social interactions [21] can improve sleep quality in older adults. Meanwhile, as technology advances, the internet has emerged as a novel platform for social interaction and information access, presenting new opportunities for sleep interventions.

According to the latest statistics from the China Internet Network Information Center, in 2024, China’s internet penetration rate reached 78%, of which one-third were aged 50 years and older [22]. As the internet penetrates further into the middle-aged and older population, increasing attention has been given to its relationship with mental health. On one hand, large-scale cohort studies have found that internet use is associated with better mental well-being [23], whereas digital exclusion is linked to a higher likelihood of depressive symptoms in older adults [24,25]. Additionally, research indicates that internet use may enhance social networks, alleviate stress, and improve depressive symptoms, cognitive function, and frailty among older adults [26-31]. Furthermore, digital medicine is increasingly adopted in health care, with the Chinese government promoting digital medical services to enhance older adults’ access to health care, sleep management, and lifestyle optimization [32]. On the other hand, multiple studies on children, adolescents, and young adults have indicated a significant association between electronic device use and sleep problems, particularly in relation to problematic screen time and internet gaming addiction [33-36]. However, the relationship between internet use and the prevalence of sleep problems among older adults remains largely unexplored, particularly in rapidly digitizing countries like China. Investigating this association holds significant practical relevance.

Given the bidirectional interactions between sleep disorders, cognitive impairment, and depressive symptoms, recent studies suggest that internet use may help improve mental health, including depressive symptoms and cognitive function. Based on this, we hypothesize that internet use may be associated with a reduced risk of sleep problems, including poor sleep quality and abnormal sleep duration, in middle-aged and older adults. To test this hypothesis, we examined both cross-sectional and longitudinal associations between internet use or internet frequency and sleep quality, as well as between internet use or internet frequency and sleep duration. This analysis was conducted using a large sample from the China Health and Retirement Longitudinal Study (CHARLS) to explore the role of the internet in sleep problems among middle-aged and older adults. Furthermore, we conducted subgroup analyses to explore whether these associations vary by sex and age.

## Methods

### Study Population

This study used data from Wave 3 in 2015 and Wave 4 in 2018 from the CHARLS dataset [37,38]. CHARLS is a national longitudinal study of middle-aged and older adults in China. Information on demographics, health, economic, and social circumstances was collected. The study design and survey instruments have been published elsewhere [39].

**Figure 1:**
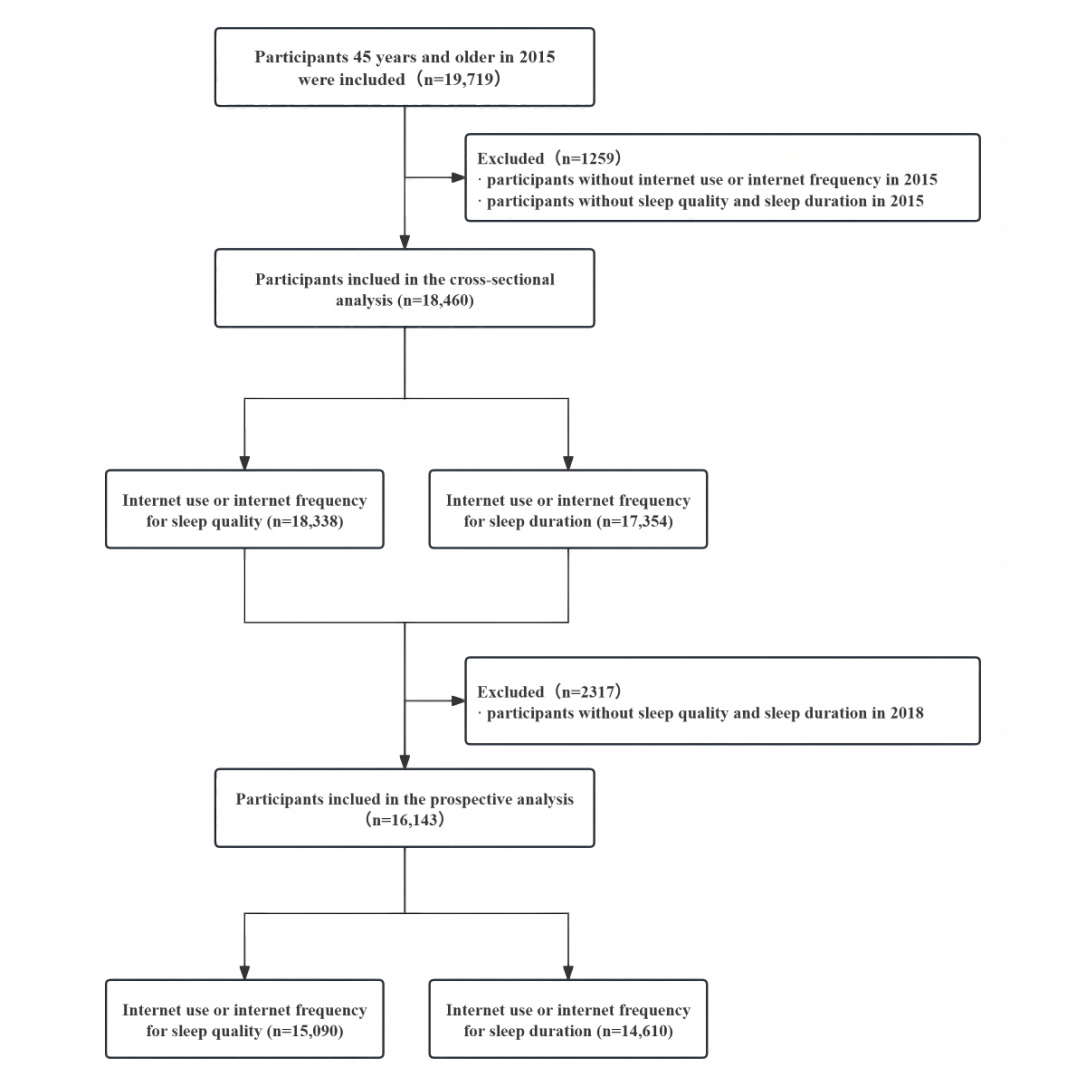
Study flow.

This study included 18,460 participants aged 45 years and older in 2015. Internet use and internet frequency were measured in 2015 (baseline), while sleep quality and duration were assessed at baseline and during the follow-up survey in 2018. Participants without internet use or internet frequency and participants without sleep quality and sleep duration in a total of 1259 were excluded from this study in 2015. In addition, 2317 participants without sleep quality and sleep duration in 2018 were excluded from the longitudinal analyses. Finally, 16,143 participants were included in the prospective analyses. A detailed selection of the study population is described in Figure 1.

### Internet

A multiple-choice question was used to determine whether participants were internet users: “Have you done any of these activities in the last month?” The options included 10 activities, such as “Used the internet,” “Interacted with friends,” and “Stock investment,” as well as option 11, “Other,” and option 12, “None of these.” Participants were classified as internet users if they selected the “Used the internet” option. Participants who selected other options but did not select “Used the internet” were classified as nonusers. Participants who did not select any option were considered to have missing data and were excluded from the analysis. For participants who indicated internet use, the internet frequency was further determined by asking the question “Frequency of activity in the last month,” which consisted of three options: “almost daily,” “almost every week,” or “not regularly.” The internet frequency access for noninternet users was defined as “never.”

### Sleep

A single question from the Center for Epidemiological Studies Depression Scale [40] was used to measure participants’ sleep quality: How often do you feel that your sleep was restless last week? Response options included rarely or none of the time (<1 day), some or a little of the time (1-2 days), occasionally or a moderate of the time (3-4 days), and most or all of the time (5-7 days). The categories of sleep quality used for analysis included “good” (<1 day; reference group), “fair” (1-2 or 3-4 days), and “poor” (5-7 days) [41].

Sleep duration was determined by the self-reported answer, “During the past month, how many hours of actual sleep did you get at night (average hours per night)?” Based on results from previous studies, 6-9 hours of sleep was beneficial for cognition in Chinese older adults [42,43]. Therefore, the study participants were categorized into three significant durations of sleep: short (<6 hours), medium (6-9 hours; reference group), and long (>9 hours).

### Covariates

Potential confounders were controlled for by evaluating the following participant characteristics: sociodemographic, lifestyle, and health characteristics at baseline, including age, sex (male or female), residence (urban or integration zone or rural or special zone), number of chronic diseases (including 14 types of chronic diseases such as hypertension, dyslipidemia, and diabetes), marital status (married or others), smoking status (never or former or current smoke), drinking status (never or former or current drink), and napping time. The sleep quality and duration in 2015 have also been included as the covariates in the longitudinal analysis. Some confounding factors, such as education, economic status, and physical activity, were excluded from the final model due to uneven sample distribution or reduced sample size (additional details regarding the covariates can be found in Multimedia Appendix 1).

### Statistical Analysis

The 2015 baseline characteristics were analyzed by stratifying participants into internet and noninternet users and by sex (male and female). Comparisons of sociodemographic, lifestyle, and health characteristics between groups were conducted using independent 2-sample *t* tests for continuous variables and chi-square tests for categorical variables. This study used a multinomial logistic regression model to estimate relative risk (RR) and 95% CIs to assess the association between baseline internet use and baseline sleep health, and its relationship with sleep status after 3 years. Covariates in the multivariate model included age, marital status, residence, chronic diseases, smoking, drinking, and napping time. Baseline sleep characteristics were additionally adjusted for in longitudinal analyses. To reduce bias and enhance the precision of the estimates, we used a weighted analysis based on individual weights, adjusted for both household and individual response rates. The cross-sectional analysis used the 2015 weights, while the longitudinal analysis applied the 2018 weights. All statistical analyses were conducted using Stata 18 (StataCorp), with a significance threshold of *P*<.05.

### Ethical Considerations

Before conducting the CHARLS survey, well-trained interviewers informed each participant about the survey’s content, and each interviewee signed an informed consent form. The survey content is strictly confidential, and all data about respondents are protected by data security and privacy laws. Images and supplementary materials ensure participant anonymity, with no identifiable information included. The Ethics Review Committee of Peking University has provided ethical approval for all waves of the CHARLS survey (approval IRB 00001052-11015).

## Results

### Baseline Characteristics of the Study Population

The baseline characteristics of the participants are listed in Table 1. The mean age was 60.0 (SD 9.8) years, and 51.5% (n=9501) were female. Among the 18,460 participants aged 45 years and older, 5111 (27.9%) participants reported fair sleep quality, and 3714 (20.3%) participants reported poor sleep quality, with a total of 8825 (48.1%) participants experiencing suboptimal sleep quality. Additionally, 5559 (32%) participants reported sleeping less than 6 hours at night, while 891 (5.1%) participants slept more than 9 hours, resulting in a total of 6450 (37.2%) participants with abnormal sleep durations. There were 1272 (6.9%) participants who reported using the internet. Among them, 74.8% (n=952) used the internet almost daily, while 14.3% (n=182) and 10.8% (n=138) of participants used it not regularly and almost weekly, respectively. The participants with internet use showed a higher ratio of good sleep quality (n=761, 60% vs n=8752, 51.3%), and a lower ratio of short (n=263, 21.3% vs n=5296, 32.9%) and long sleep duration (n=21, 1.7% vs n=870, 5.4%) than those without internet. Compared to noninternet participants, internet participants were younger, more likely to be male, resided in urban areas, had a lower burden of chronic disease, and were more likely to smoke and drink alcohol; the latter behaviors may be associated with the higher proportion of male internet users.

**Table 1:** Participant characteristics by internet use at baseline in the China Health and Retirement Longitudinal Study, 2015.

Characteristic		Overall (n=18,460)	Internet nonuse (n=17,188)	Internet use (n=1272)	*P* value	
Age, (years), mean (SD)		60.0 (9.8)	60.5 (9.8)	53.4 (7.4)	<.001^a^	
**Age (years), n (%)**						<.001^a^
	45-59	9244 (50.1)	8230 (47.9)	1014 (79.7)		
	60-79	8582 (46.5)	8328 (48.5)	254 (20)		
	≥80	634 (3.4)	630 (3.7)	4 (0.3)		
**Sex, n (%)**						<.001^a^
	Male	8959 (48.5)	8175 (47.6)	784 (61.6)		
	Female	9501 (51.5)	9013 (52.4)	488 (38.4)		
**Marital status, n (%)**						<.001^a^
	Married	16,095 (87.2)	14,910 (86.7)	1185 (93.2)		
	Others	2365 (12.8)	2278 (13.3)	87 (6.8)		
**Residence, n (%)**						<.001^a^
	Urban	2519 (14)	1955 (11.6)	564 (47.9)		
	Intergration zone	827 (4.6)	726 (4.3)	101 (8.6)		
	Rural	14,537 (80.9)	14,034 (83.6)	503 (42.7)		
	Special zone	77 (0.4)	68 (0.4)	9 (0.8)		
Chronic diseases, mean (SD)		2.0 (2.7)	2.0 (2.7)	1.4 (2.3)	<.001^a^	
**Smoke, n (%)**						.006^a^
	Never smoked	10,810 (58.7)	10,119 (59.0)	691 (54.5)		
	Former smoker	1944 (10.6)	1789 (10.4)	155 (12.2)		
	Current smoker	5668 (30.8)	5247 (30.6)	421 (33.2)		
**Drink, n (%)**						<.001^a^
	Never drink	9772 (53)	9335 (54.4)	437 (34.4)		
	Former drinker	2045 (11.1)	1941 (11.3)	104 (8.2)		
	Current drinker	6623 (35.9)	5895 (34.3)	728 (57.4)		
Napping time (minutes), mean (SD)		38.9 (44.9)	39.0 (45.3)	36.5 (38.0)	.06	
**Sleep quality, n (%)**						<.001^a^
	Good	9513 (51.9)	8752 (51.3)	761 (60)		
	Fair	5111 (27.9)	4759 (27.9)	352 (27.7)		
	Poor	3714 (20.3)	3558 (20.8)	156 (12.3)		
Sleep duration (hours), mean (SD)		6.4 (1.9)	6.4 (2.0)	6.5 (1.3)	<.001^a^	
**Sleep duration (hours), n (%)**						<.001^a^
	<6	5559 (32)	5296 (32.9)	263 (21.3)		
	6-9	10,904 (62.8)	9954 (61.7)	950 (77)		
	>9	891 (5.1)	870 (5.4)	21 (1.7)		
**Internet frequency, n (%)**						<.001^a^
	Never	17,185 (93.1)	17,185 (100)	0 (0)		
	Not regularly	184 (1)	2 (<1)	182 (14.3)		
	Almost every week	138 (0.7)	0 (0.0)	138 (10.8)		
	Almost daily	953 (5.2)	1 (<1)	952 (74.8)		

^a^*P*<.05.

### Cross-Sectional Association Between Internet and Sleep

Table 2 presents the associations between baseline internet use or internet frequency and baseline sleep quality. Internet use was significantly associated with both fair and poor sleep quality. After adjustment, the RR of poor sleep quality remained significantly lower among internet users than nonusers (RR 0.64, 95% CI 0.46-0.89). Notably, a consistent association was observed across different frequencies of internet use. Specifically, compared to those who did not use the internet in the past month, almost daily internet users showed a significantly lower RR of fair sleep quality (RR 0.71, 95% CI 0.54-0.94) and poor sleep quality (RR 0.67, 95% CI 0.46-0.97) after adjustment.

Table 3 shows the associations between internet use or internet frequency and sleep duration. Internet use was significantly associated with both short and long sleep durations. After adjustment, internet users had a significantly lower RR of short sleep duration (RR 0.69, 95% CI 0.49-0.96) and long sleep duration (RR 0.28, 95% CI 0.16-0.48) compared to noninternet users. Similarly, internet frequency use was significantly associated with reduced RR of both short and long sleep durations. After further adjustment, the RR of long sleep duration remained significantly lower among almost daily users (RR 0.22, 95% CI 0.11-0.44) compared to those who did not use the internet in the last month.

**Table 2:** Cross-sectional associations between baseline internet use or internet frequency and sleep quality in 2015.

Outcomes				Unadjusted model (n=18,083)				Adjusted model^a^ (n=17,428)		
				RR^b^ (95% CI)		*P* value		RR (95% CI)		*P* value
**Internet use and sleep quality**										
	Good			1 (reference)		N/A^c^		1 (reference)		N/A
	Fair			0.70 (0.56-0.87)		.002^d^		0.81 (0.64-1.02)		.08
	Poor			0.46 (0.34-0.61)		<.001^d^		0.64 (0.46-0.89)		.008^d^
**Internet frequency and sleep quality**										
	Good			1 (reference)		N/A		1 (reference)		N/A
	**Fair**									
		Never	1 (reference)		N/A		1 (reference)		N/A	
		Not regularly	1.11 (0.69-1.80)		.67		1.20 (0.75-1.93)		.46	
		Almost every week	1.16 (0.74-1.83)		.51		1.31 (0.81-2.10)		.27	
		Almost daily	0.61 (0.47-0.80)		<.001^d^		0.71 (0.54-0.94)		.02^d^	
	**Poor**									
		Never	1 (reference)		N/A		1 (reference)		N/A	
		Not regularly	0.50 (0.29-0.87)		.02^d^		0.50 (0.28-0.90)		.02^d^	
		Almost every week	0.45 (0.25-0.81)		.008^d^		0.57(0.30-1.07)		.08	
		Almost daily	0.45 (0.32-0.64)		<.001^d^		0.67(0.46-0.97)		.04^d^	

^a^Adjusted for age, sex, marital status, residence, chronic diseases, smoking status, drinking status, and napping time.

^b^RR: relative risk.

^c^Not applicable.

^d^*P*<.0*5*.

**Table 3:** Cross-sectional associations between baseline internet use or internet frequency and sleep duration in 2015.

Outcomes				Unadjusted model (n=17,109)					Adjusted model^a^ (n=16,515)		
				RR^b^ (95% CI)		*P* value		RR (95% CI)			*P* value
**Internet use and sleep duration (hours)**											
	<6		0.55 (0.41-0.75)		<.001^c^		0.69 (0.49-0.96)			.03^c^	
	6-9		1 (reference)		N/A^d^		1 (reference)			N/A	
	>9		0.16 (0.09-0.30)		<.001^c^		0.28 (0.16-0.48)			<.001^c^	
**Internet frequency and sleep duration**											
	6-9 hours		1 (reference)		N/A		1 (reference)			N/A	
	**<6 hours**										
		Never	1 (reference)		N/A		1 (reference)			N/A	
		Not regularly	0.57 (0.27-1.22)		.15		0.75 (0.31-1.81)			.53	
		Almost every week	0.49 (0.31-0.79)		*.*004^c^		0.65 (0.39-1.07)			.09	
		Almost daily	0.55 (0.39-0.79)		.001^c^		0.68 (0.46-1.00)			.05	
	**>9 hours**										
		Never	1 (reference)		N/A		1 (reference)			N/A	
		Not regularly	0.24 (0.08-0.71)		.01^c^		0.43 (0.14-1.26)			.13	
		Almost every week	0.29 (0.10-0.81)		*.*02^c^		0.53 (0.19-1.49)			.23	
		Almost daily	0.14 (0.06-0.30)		<.001^c^		0.22 (0.11-0.44)			<.001^c^	

^a^Adjusted for age, sex, marital status, residence, chronic diseases, smoking status, drinking status, and napping time.

^b^RR: relative risk.

^c^*P*<.05.

^d^Not applicable.

### Longitudinal Association Between Internet and Sleep

Table 4 shows the association between baseline internet use or internet frequency and sleep quality over a 3-year follow-up. Baseline internet use was associated with a reduced risk of fair (RR 0.66, 95% CI 0.51-0.86) and poor sleep quality (RR 0.60, 95% CI 0.44-0.81), even after adjustment, compared to nonusers. Similarly, the frequency of internet use showed consistent results: participants who used the internet almost daily had a significantly lower risk of fair sleep quality (RR 0.57, 95% CI 0.43-0.74) compared to those who had not used the internet in the past month. Additionally, those who used the internet almost weekly or daily had a significantly lower risk of poor sleep quality, with adjusted RRs of 0.25 (95% CI 0.12-0.54) and 0.59 (95% CI 0.41-0.84), respectively, compared to nonusers.

Table 5 summarizes the relationship between baseline internet use or internet frequency and sleep duration over 3 years. Compared to baseline noninternet users, internet users had a significantly lower risk of both short (RR 0.73, 95% CI 0.53-1.00) and long sleep durations (RR 0.39, 95% CI 0.21-0.72). Similar trends were observed for internet frequency. Compared to those who had not used the internet in the past month, almost weekly, and daily internet users had significantly reduced RR of both short and long sleep durations. After adjustment, the reduced RR of long sleep duration remained significant among almost daily internet users (RR 0.32, 95% CI 0.15-0.69) compared to nonusers.

**Table 4:** Longitudinal associations between baseline internet use or internet frequency and sleep quality in 2018.

Outcomes				Unadjusted model (n=14,880)				Adjusted model^a^ (n=14,461)		
				RR^b^ (95% CI)		*P* value		RR (95% CI)		*P* value
**Internet use and sleep quality**										
	Good		1 (reference)		N/A^c^		1 (reference)		NA	
	Fair		0.70 (0.55-0.88)		.003^d^		0.66 (0.51-0.86)		.002^d^	
	Poor		0.49 (0.37-0.65)		<.001^d^		0.60 (0.44-0.81)		.001^d^	
**Internet frequency and sleep quality**										
	Good		1 (reference)		N/A		1 (reference)		N/A	
	**Fair**									
		Never	1 (reference)		N/A		1 (reference)		N/A	
		Not regularly	1.42 (0.72-2.80)		.32		1.51 (0.70-3.23)		.29	
		Almost every week	0.64 (0.38-1.09)		.10		0.64 (0.36-1.12)		.12	
		Almost daily	0.62 (0.48-0.80)		<.001^d^		0.57 (0.43-0.74)		<.001^d^	
	**Poor**									
		Never	1 (reference)		N/A		1 (reference)		N/A	
		Not regularly	0.73 (0.40-1.33)		.29		1.05 (0.57-1.95)		.87	
		Almost every week	0.22 (0.11-0.45)		<.001^d^		0.25 (0.12-0.54)		<.001^d^	
		Almost daily	0.49 (0.36-0.68)		<.001^d^		0.59 (0.41-0.84)		.003^d^	

^a^Adjusted for age, sex, marital status, residence, chronic diseases, smoking status, drinking status, napping time, and baseline sleep.

^b^RR: relative risk.

^c^Not applicable.

^d^*P*<.05.

**Table 5:** Longitudinal associations between baseline internet use or internet frequency and sleep duration in 2018.

Outcomes				Unadjusted model (n=14,403)					Adjusted model^a^ (n=14,000)		
				RR^b^ (95% CI)		*P* value		RR (95% CI)			*P* value
**Internet use and sleep duration (hours)**											
	<6			0.56 (0.41-0.78)		.001^c^		0.73 (0.53-1.00)			.047^c^
	6-9			1 (reference)		N/A^d^		1 (reference)			N/A
	>9			0.35 (0.13-0.92)		.03^c^		0.39 (0.21-0.72)			.003^c^
**Internet frequency and sleep duration**											
	6-9 hours			1 (reference)		N/A		1 (reference)			N/A
	**<6 hours**										
		Never	1 (reference)		N/A		1 (reference)			N/A	
		Not regularly	0.60 (0.28-1.30)		.19		0.71 (0.33-1.51)			.37	
		Almost every week	0.58 (0.37-0.93)		.03^c^		0.73 (0.45-1.18)			.20	
		Almost daily	0.56 (0.38-0.82)		.003^c^		0.74 (0.51-1.06)			.10	
	**>9 hours**										
		Never	1 (reference)		N/A		1 (reference)			N/A	
		Not regularly	0.58 (0.21-1.54)		.27		1.02 (0.38-2.75)			0.969	
		Almost every week	0.08 (0.01-0.58)		.01^c^		0.15 (0.02-1.10)			.06	
		Almost daily	0.35 (0.11-1.16)		.09		0.32 (0.15-0.69)			.004^c^	

^a^Adjusted for age, sex, marital status, residence, chronic diseases, smoking status, drinking status, napping time, and baseline sleep.

^b^RR: relative risk.

^c^*P*<.05.

^d^N/A: not applicable.

### Longitudinal Association Between Internet and Sleep of Different Sexes or Ages

The subgroup analyses presented that male participants were more likely to use the internet than female participants (n=643, 8.3% vs n=405, 4.8%) at baseline, as well as to use it more frequently (not regularly: n=99, 1.3% vs n=54, 0.6%; almost every week: n=86, 1.1% vs n=33, 0.4%; almost daily: n=459, 5.9% vs n=318, 3.8%). Regarding sleep performance, both 2015 and 2018 data showed that sleep quality was significantly better for male participants than female participants (2018: good: n=3958, 54.5% vs n=2996, 38%; fair: n=2052, 28.2% vs n=2708, 34.4%; poor: n=1259, 17.3% vs n=2171, 27.6%). Additionally, moderate sleep duration prevalence was higher for male participants than female participants in 2015 and 2018 (2018: 6- to 9-hour sleep duration: n=4705, 63.5% vs n=4277, 53.1%; Table S1 in Multimedia Appendix 1). Compared to female participants, male participants were older, more likely to be married, had a higher burden of chronic diseases, spent more time napping, and were more likely to smoke and drink alcohol.

In the adjusted logistic models, internet users exhibited a significantly reduced RR of poor sleep after 3 years compared to noninternet users, with a greater risk reduction observed in female participants (RR 0.57, 95% CI 0.36-0.89) than in male participants (RR 0.61, 95% CI 0.40-0.92). A similar significant association was found between internet frequency and sleep quality, with both fair and poor sleep quality showing more pronounced effects in daily internet users compared to noninternet users in both female (fair: RR 0.52, 95% CI 0.36-0.74; poor: RR 0.58, 95% CI 0.35-0.95) and male (fair: RR 0.61, 95% CI 0.41-0.89; poor: RR 0.60, 95% CI 0.36-0.98) participants. However, the relationship between internet use and sleep duration showed the opposite pattern between the sexes. Specifically, significant associations were found only in male participants, and exclusively for the risk of long sleep duration, with internet users (RR 0.33, 95% CI 0.14-0.76) and daily internet users (RR 0.30, 95% CI 0.11-0.78) showing a lower risk compared to nonusers.

In the age subgroup analysis, most internet users were concentrated in the 45- to 59-year age group, accounting for 79.7% (n=1014) of the internet users in the study. This group’s internet use was significantly associated with a reduced risk of poor sleep quality (RR 0.64, 95% CI 0.44-0.92), short sleep duration (RR 0.69, 95% CI 0.48-1.00), and long sleep duration (RR 0.36, 95% CI 0.18-0.74) compared to noninternet users, in the adjusted logistic regression model. Compared to nonusers, daily internet users showed a reduced risk of fair sleep quality (RR 0.69, 95% CI 0.52-0.93), poor sleep quality (RR 0.62, 95% CI 0.40-0.95), and long sleep duration (RR 0.38, 95% CI 0.16-0.90). A similar protective effect of internet use on sleep outcomes was observed in the 60- to 79-year age group. However, the >80-year age group was excluded from the analysis due to this subgroup’s minimal proportion of internet users (n=4, 0.3%; detailed results are presented in Tables S1-S3 in Multimedia Appendix 1).

## Discussion

### Principal Findings

To our knowledge, this study was the first analysis to examine the association between internet use and sleep problems among middle-aged and older adults. The results of our study revealed a significant association between internet use and a decreased likelihood of experiencing poor sleep quality, as well as the higher internet frequency decreasing the incidence risk of short and long sleep duration, especially almost daily use of the internet.

With the increasing popularity of information and communication technology, the internet penetration rate among middle-aged and older adults in China has proliferated in the last decade, with the group of internet users aged 50 years and older rising from 6.7% in 2015 [44] to 33.3% in 2024 [22], as reported by the China Internet Network Information Center. The data in CHARLS shows the same trend, with the proportion of middle-aged and older people using the internet rising from 6.9% in 2015 to 40.7% in 2020 [45].

As an intrusive factor significantly influencing lifestyle, numerous studies have explored the relationship between internet use and sleep, predominantly focusing on children, adolescents, and young adults. This focus arises from historical trends, as adolescents, especially those prone to internet addiction, represented a major user group. Studies consistently link digital media use in these populations to shorter sleep duration and poorer sleep quality [46-48]. However, the rapid proliferation of internet use among middle-aged and older adults in China, mainly through platforms such as WeChat, digital payment systems, and short video apps, has introduced new dynamics to this relationship. Sleep quality inherently varies considerably across different age groups, and the effects of internet use on sleep are not uniform. Contrary to the predominantly negative effects observed in children and adolescents, this study is the first to identify a positive association between appropriate internet use and improved sleep quality in middle-aged and older adults. This highlights the nuanced interplay between digital technology and lifestyle outcomes in different age demographics.

Both physiological health and psychological health have significant biphasic effects on sleep [49,50]. This study concluded that appropriate internet use is associated with a reduced risk of poor sleep among middle-aged and older adults. This relationship is likely attributable to the positive impact of appropriate internet use on middle-aged and older individuals’ physiological and psychological well-being. First, appropriate internet use improves sleep by reducing depressed mood in middle-aged and older adults. A recent study revealed a significant association between internet exclusion and a higher likelihood of experiencing depression in older adults [25]. At the same time, the study also supports that internet use was associated with fewer depressive symptoms and a higher internet frequency was related to better mental health in China [23]. Internet use not only enhances the ability of seniors to stay in touch with family and friends (video chatting, eg, WeChat) but also enhances social network support for seniors, increases the frequency of social interactions, reduces the risk of loneliness, and socialization, which in turn reduces the risk of depression and improves sleep [25,26,28,31,51]. Second, lack of employment leads to loss of social roles and self-efficacy among older adults. Studies have shown that internet use can reduce stress among older persons experiencing resource loss and gain and can also help to improve cognitive functioning among middle-aged and older persons [27-29], which can help older persons regain opportunities to participate in recreational life and employment, increase self-efficacy, reduce emptiness and anxiety, and help to improve sleep [50]. Third, using internet helps the older adults by providing them with more convenient data, medication insurance, and acceptance of health knowledge. Via the internet, older adults can access and follow the latest information in health management and purchase medication and health devices, preventing frailty [31] and functional dependency [24]. Moreover, internet-mediated interventions demonstrate significant potential for delivering effective lifestyle programs for older adults [52]. This contributes to improved medical health and related anxiety, improving sleep problems among middle-aged and older adults.

The sex subgroup analysis of this study found that middle-aged and older men performed better than women in terms of sleep quality and duration, which is consistent with the results of the previous survey on sleep in China [53,54]. Sleep disturbances are common in older women, affecting >40% to 60% of perimenopausal or postmenopausal women [55]. The changes in estrogen levels can result in hot flashes, night sweats, headaches, and palpitations, which can directly affect sleep, increase the risk for sleep apnea, and cause mood changes, especially depression [56,57]. The sex-specific subgroup analysis aligned with the overall findings, indicating that both male and female internet users exhibited a lower risk of sleep problems compared to noninternet users. These results suggest that promoting internet access may be a viable strategy to improve sleep outcomes among both middle-aged and older men and women. The age subgroup analysis of this study suggests that those who use the internet are mainly in the younger age group. This is the same as the age structure of Chinese internet users in 2015, which reported that 17.3% were aged 40>age>59 years and 2.4% were aged >60 [26]. Internet use reduces the risk of poorer sleep than internet nonusers, which is consistent with the results of the ungrouped analysis.

The relationship between internet use and sleep problems is likely bidirectional, influenced by use patterns, devices, content, and timing [47]. Studies have found a strong connection between bedtime internet use and poor sleep quality. Using the internet before sleep can increase mental arousal, making it harder to fall asleep [48,58,59]. Blue light causes behavioral arousal, elevating corticosterone and delaying sleep onset [60]. Additionally, research indicates that portable devices (such as smartphones and tablets) are more strongly associated with poor sleep quality when used before bed than traditional devices [46,47,61]. The interactivity of internet content is also a significant factor, with screen-based engagement like internet gaming, multitasking, and social media participation being more likely to exacerbate sleep problems [48,62-65]. More importantly, while internet use directly impacts sleep quality, it may also serve as a coping mechanism for individuals with insomnia. Many people turn to social media to alleviate anxiety or negative emotions during periods of poor sleep, particularly under stressful conditions such as the COVID-19 pandemic [66,67]. This study provides a preliminary analysis of the issue. We did not observe a significant increase in internet frequency among older adults with poor sleep quality in 2015 by 2018 (Table S5 in Multimedia Appendix 1), possibly due to these individuals’ more incredible difficulty in accessing and adapting to internet use at baseline. Future research should adopt more rigorous designs, such as randomized controlled trials or longitudinal studies, incorporating data from multiple time points and using mediation or path analysis to explore internet user’s impact on sleep. In summary, more rigorous longitudinal and experimental studies are needed to accurately assess the effects of internet use on sleep, particularly those that manipulate digital media use. Clinical psychiatrists’ involvement is crucial, as sleep issues often require psychological and pharmacological treatments, which need to be controlled. Although current research shows a positive trend in internet use among older adults, particularly in reducing anxiety and depression, the risk of internet addiction remains a concern. With the advancement of artificial intelligence, the internet can meet more mental health needs, and internet addiction may affect all age groups. Maintaining a balanced digital life is no longer just an adolescent issue [68]. Therefore, clinical recommendations should remain cautious, focusing on current findings and suggesting future research directions. This study suggests that older adults who do not use the internet should gradually increase their use, avoiding internet use before bedtime. Clinical interventions should be personalized to prevent the adverse effects of excessive use while leveraging the benefits of the internet to promote sleep health.

### Limitations

Although this study is the first to explore the relationship between internet and sleep among middle-aged and older adults using a large, nationally representative cohort with both cross-sectional and longitudinal analyses, several evident limitations remain, primarily due to gaps in objective data. First, some key survey items and related factors within the cohort require further refinements, such as internet use duration, screen time before bed, comprehensive sleep assessment tools (eg, Pittsburgh Sleep Quality Index), continuous sleep tracking, and access to health care. Second, due to the current stage of internet use in China, the analysis of specific subgroups could not be deeply explored. For example, there is limited data on internet use among individuals older than 80 years of age, and similarly, with a relatively more minor number of internet users compared to nonusers, further subgroup analyses and the inclusion of certain covariates, such as socioeconomic status data and imbalanced education sample distribution, are also challenging. These issues are primarily due to the rapid spread of the internet in China, and addressing them will take time. Third, the observed cohort cannot be definitively established as causal due to insufficient data. Future studies will address this issue by incorporating data from multiple time points and using methods such as mediation or path analysis or using randomized controlled trials or alternative study designs to establish the causal or bidirectional relationship between internet use and sleep among middle-aged and older adults. Finally, the complex effects of COVID-19 on sleep and internet use made it difficult to incorporate the most recent 2020 CHARLS data into this study. As a result, data that could have helped further elucidate the diversity and complexity of internet use, such as the content and tools used digitally, were excluded from the analysis. To address all the aforementioned limitations, we plan to further explore these issues in future studies by continuing to track the latest data from this cohort, particularly data collected during the non–COVID-19 period, or by integrating cross-national cohort data.

### Conclusions

This study identified a significant association between internet and sleep among middle-aged and older adults, revealing that regular internet users, particularly those engaging in almost daily use, exhibited a lower likelihood of poor sleep quality and excessive sleep duration. To mitigate sleep-related issues, it is essential to develop effective interventions that promote appropriate and active participation of middle-aged and older adults in the digital society, specifically encouraging consistent and balanced internet use.

## Appendix

Multimedia Appendix 1 Additional files.

## Abbreviations

CHARLS: China Health and Retirement Longitudinal Study
RR: relative risk

## Data Availability

The data that support the findings of this study are available from the China Health and Retirement Longitudinal Study data repository.
