# Supplementary material for: Association Between Internet Use and Sleep Health Among Middle-Aged and Older Chinese Individuals: Nationwide Longitudinal Study
Source: J Med Internet Res. 2025 Apr 16;27:e71030. doi: 10.2196/71030 (PMC12044320; doi:10.2196/71030)
Supplement: Multimedia Appendix 1 [file jmir_v27i1e71030_app1.docx]

## Multimedia Appendix 1

## Covariates：

The residence item has seven options, “urban” means option1 (main city zone), “integration zone” means urban-rural2 (combination zone between urban and rural areas), “rural” includes option3(the town center), option4 (ZhenXiang area), option6(Township central) and option7(Village); “Special zone” means option5. All the chronic diseases include hypertension, dyslipidemia, diabetes/high blood sugar, cancer/malignant, chronic lung diseases, liver disease, heart attack, heart attack / coronary heart disease/ angina /congestive heart failure /other heart problems, stroke, kidney disease, stomach/other digestive disease, emotional/nervous/psychiatric problems, memory-related disease, arthritis /rheumatism, asthma). Marital status is described as “married” and “others”. “married” includes option1 (Married with spouse present) and option2 (Married but not living with spouse temporarily for reasons such as work); “others” include option3 (Separated), option4 (Divorced), option5 (Widowed), option6 (Never married) and option7 (Cohabitated).

## As mentioned in the Methods section under the covariates, some confounding factors were excluded from the final model due to issues with sample distribution or reduced sample size. Specifically, education level distribution was highly uneven (see Table S4). When attempting to include economic status through per capita household income, we observed a substantial reduction in sample size, with only half of the participants (n=7,572) remaining after inclusion in the multivariate model. A similar issue occurred with the inclusion of physical activity, with the sample size decreasing to n=7,246.

## Table S1. Participant characteristics by gender use at baseline in the China Health and Retirement Longitudinal Study, (2015-2018, n =16143)

| **Characteristic** | **Overall**  n=16143 | **male**  n=7733 | **female**  n=8410 | **P value** |
| --- | --- | --- | --- | --- |
| Age, (years) , mean (SD) | 59.8 (9.6) | 60.0 (9.6) | 59.5 (9.6) | <.001 ^a^ |
| Age, n (%) |  |  |  | 0.14 |
| 45-59 | 8188 (50.7) | 3859 (49.9) | 4329 (51.5) |  |
| 60-79 | 7491 (46.4) | 3649 (47.2) | 3842 (45.7) |  |
| ≥80 | 464 (2.9) | 225 (2.9) | 239 (2.8) |  |
| Marital Status, n (%) |  |  |  | <.001 ^a^ |
| Married | 14204 (88.0) | 7078 (91.5) | 7126 (84.7) |  |
| Others | 1939 (12.0) | 655 (8.5) | 1284 (15.3) |  |
| Residence, n (%) |  |  |  | 0.75 |
| Urban | 2009 (12.7) | 953 (12.6) | 1056 (12.8) |  |
| Intergration zone | 695 (4.4) | 345 (4.5) | 350 (4.3) |  |
| Rural | 13042 (82.5) | 6255 (82.5) | 6787 (82.5) |  |
| Special Zone | 65 (0.4) | 33 (0.4) | 32 (0.4) |  |
| Chronic diseases^a^ | 1.9±2.7 | 1.9±2.8 | 1.9±2.6 | <.001 ^a^ |
| Smoke, n (%) |  |  |  | <.001 ^a^ |
| Never smoke | 9536 (59.2) | 1750 (22.7) | 7786 (92.8) |  |
| Former smoke | 1655 (10.3) | 1492 (19.3) | 163 (1.9) |  |
| Current smoke | 4919 (30.5) | 4475 (58.0) | 444 (5.3) |  |
| Drink, n (%) |  |  |  | <.001 ^a^ |
| Never drink | 8598 (53.3) | 2065 (26.7) | 6533 (77.8) |  |
| Former drink | 1722 (10.7) | 1109 (14.4) | 613 (7.3) |  |
| Current drink | 5804 (36.0) | 4548 (58.9) | 1256 (14.9) |  |
| Napping time, (minutes) , mean (SD) | 38.7 (44.8) | 43.5 (45.6) | 34.2 (43.6) | <.001 ^a^ |
| Sleep quality 2015, n (%) |  |  |  | <.001 ^a^ |
| Good | 8355 (52.0) | 4657 (60.5) | 3698 (44.2) |  |
| Fair | 4480 (27.9) | 1877 (24.4) | 2603 (31.1) |  |
| Poor | 3227 (20.1) | 1166 (15.1) | 2061 (24.6) |  |
| Sleep duration 2015, (hours)^a^, mean (SD) | 6.4 (1.9) | 6.5 (1.8) | 6.2 (2.1) | <.001 ^a^ |
| Sleep duration 2015, n (%) |  |  |  | <.001 ^a^ |
| <6h | 4875 (32.1) | 1983 (26.9) | 2892 (36.9) |  |
| 6-9h | 9553 (62.9) | 5005 (68.0) | 4548 (58.1) |  |
| >9h | 767 (5.0) | 377 (5.1) | 390 (5.0) |  |
| Sleep quality 2018, n (%) |  |  |  | <.001 ^a^ |
| Good | 6954 (45.9) | 3958 (54.5) | 2996 (38.0) |  |
| Fair | 4760 (31.4) | 2052 (28.2) | 2708 (34.4) |  |
| Poor | 3430 (22.6) | 1259 (17.3) | 2171 (27.6) |  |
| Sleep duration 2018, (hours) , mean (SD) | 6.2 (2.0) | 6.4 (1.9) | 6.0 (2.1) | <.001 ^a^ |
| Sleep duration 2018, n (%) |  |  |  | <.001 ^a^ |
| <6h | 5686 (36.8) | 2303 (31.1) | 3383 (42.0) |  |
| 6-9h | 8982 (58.1) | 4705 (63.5) | 4277 (53.1) |  |
| >9h | 790 (5.1) | 399 (5.4) | 391 (4.9) |  |
| internet |  |  |  | <.001 ^a^ |
| No | 15095 (93.5) | 7090 (91.7) | 8005 (95.2) |  |
| Yes | 1048 (6.5) | 643 (8.3) | 405 (4.8) |  |
| Internet frequency |  |  |  | <.001 ^a^ |
| Never | 15094 (93.5) | 7089 (91.7) | 8005 (95.2) |  |
| Not regularly | 153 (0.9) | 99 (1.3) | 54 (0.6) |  |
| Almost every week | 119 (0.7) | 86 (1.1) | 33 (0.4) |  |
| Almost daily | 777 (4.8) | 459 (5.9) | 318 (3.8) |  |

^a^ *P*<.05

## Table S2. Longitudinal predictions of sleep in 2018 by internet in 2015 of different sexes（Adjusted Model）

| **Outcomes** | **male**^a^ | | **female**^a^ | |
| --- | --- | --- | --- | --- |
|  | **RR**^b^ **(95%CI)** | **P Value** | **RR (95%CI)** | **P Value** |
| **Internet use-Sleep quality** |  |  |  |  |
| Total Number | 6976 |  | 7485 |  |
| Good | 1(Reference) | N/ A^c^ | 1(Reference) | N/ A |
| Fair | 0.73(0.50,1.07) | .11 | 0.57(0.41,0.80) | .001^d^ |
| Poor | 0.61(0.40,0.92) | .02^d^ | 0.57(0.36,0.89) | .01^d^ |
| **Internet frequency-Sleep quality** |  |  |  |  |
| Good | 1 (Reference) | N/ A | 1 (Reference) | N/ A |
| Fair |  |  |  |  |
| never | 1 (Reference) | N/ A | 1 (Reference) | N/ A |
| not regularly | 1.48(0.52,4.17) | .46 | 1.60(0.76,3.34) | .21 |
| almost every week | 0.80(0.41,1.54) | .50 | 0.40(0.15,1.07) | .07 |
| almost daily | 0.61(0.41,0.89) | .01^d^ | 0.52(0.36,0.74) | <.001^d^ |
| Poor |  |  |  |  |
| never | 1(Reference) | NA | 1(Reference) | NA |
| not regularly | 0.96(0.46,1.99) | .91 | 1.20(0.46,3.11) | .71 |
| almost every week | 0.40(0.16,0.94) | .04^d^ | 0.08(0.02,0.37) | .001^d^ |
| almost daily | 0.60(0.36,0.98) | .04^d^ | 0.58(0.35,0.95) | .03^d^ |
| **Internet use-Sleep duration** |  |  |  |  |
| Total Number | 6808 |  | 7192 |  |
| <6h | 0.72(0.45,1.13) | .16 | 0.74(0.54,1.02) | .06 |
| 6-9h | 1(Reference) | NA | 1(Reference) | N/ A |
| >9h | 0.33(0.14,0.76) | .009^d^ | 0.51(0.20,1.28) | .15 |
| **Internet frequency-Sleep duration** |  |  |  |  |
| 6-9h | 1 (Reference) | N/ A | 1 (Reference) | N/ A |
| <6h |  |  |  |  |
| never | 1 (Reference) | N/ A | 1 (Reference) | N/ A |
| not regularly | 0.65(0.24,1.77) | .40 | 0.82(0.34,1.94) | .65 |
| almost every week | 0.66(0.37,1.19) | .17 | 0.90(0.38,2.14) | .81 |
| almost daily | 0.74(0.43,1.30) | .30 | 0.72(0.51,1.02) | .07 |
| >9h |  |  |  |  |
| never | 1 (Reference) | N/ A | 1 (Reference) | N/ A |
| not regularly | 0.986(0.26,3.77) | .98 | 1.26(0.31,5.08) | .75 |
| almost every week | N/ A | <0.001^d^ | 0.63(0.09,4.22) | .63 |
| almost daily | 0.30(0.11,0.78) | .01^d^ | 0.37(0.11,1.27) | .11 |

^a^Adjusted for age, marital status, residence, chronic diseases, smoking status, drinking status, napping time.

^b^RR: relative risk.

^c^Not applicable.

^d^*P*<.0*5*

## Table S3. Longitudinal predictions of sleep in 2018 by internet in 2015 of different age groups（45-59，60-79，Adjusted Model）

| **Outcomes** | **45-59**^a^ | | **60-79**^a^ | |
| --- | --- | --- | --- | --- |
|  | **RR**^b^ **(95%CI)** | **P Value** | **RR (95%CI)** | **P Value** |
| **Internet use-Sleep quality** |  |  |  |  |
| Total Number | 7437 |  | 6732 |  |
| Good | 1 (Reference) | N/ A^c^ | 1 (Reference) | N/ A |
| Fair | 0.79(0.59,1.07) | .13 | 0.39(0.22,0.66) | .001^d^ |
| Poor | 0.64(0.44,0.92) | .02^d^ | 0.49(0.27,0.90) | .02^d^ |
| **Internet frequency-Sleep quality** |  |  |  |  |
| Good | 1 (Reference) | N/ A | 1 (Reference) | N/ A |
| Fair |  |  |  |  |
| never | 1 (Reference) | NA | 1 (Reference) | NA |
| not regularly | 1.75(0.70,4.40) | .23 | 1.01(0.39,2.62) | .98 |
| almost every week | 0.71(0.39,1.29) | .26 | 0.47(0.10,2.20) | .34 |
| almost daily | 0.69(0.52,0.93) | .01^d^ | 0.30(0.16,0.57) | <0.001^d^ |
| Poor |  |  |  |  |
| never | 1 (Reference) | N/ A | 1 (Reference) | N/ A |
| not regularly | 1.27(0.63,2.56) | .50 | 0.51(0.11,2.36) | .39 |
| almost every week | 0.29(0.13,0.67) | .004^d^ | NA | <0.001^d^ |
| almost daily | 0.62(0.40,0.95) | .03^d^ | 0.52(0.27,1.00) | .05 |
| **Internet use-Sleep duration** |  |  |  |  |
| Total Number | 7135 |  | 6496 |  |
| <6h | 0.69(0.48,1.00) | 0.05 | 0.71(0.47,1.09) | .12 |
| 6-9h | 1 (Reference) | N/ A | 1 (Reference) | N/ A |
| >9h | 0.36(0.18,0.74) | .005^d^ | 0.57(0.18,1.73) | .32 |
| **Internet frequency-Sleep duration** |  |  |  |  |
| 6-9h | 1 (Reference) | N/ A | 1 (Reference) | N/ A |
| <6h |  |  |  |  |
| never | 1 (Reference) | N/ A | 1 (Reference) | N/ A |
| not regularly | 0.47(0.20,1.14) | .10 | 1.96(0.72,5.32) | .19 |
| almost every week | 0.79(0.46,1.33) | .38 | 0.27(0.08,0.92) | .04^d^ |
| almost daily | 0.72(0.46,1.13) | .15 | 0.63(0.39,0.99) | .05 |
| >9h |  |  |  |  |
| never | 1(Reference) | N/ A | 1(Reference) | N/ A |
| not regularly | 0.38(0.09,1.63) | .19 | 4.65(1.19,18.18) | .03^d^ |
| almost every week | 0.20(0.03,1.39) | .10 | NA | <0.001^d^ |
| almost daily | 0.38(0.16,0.90) | .03^d^ | 0.27(0.05,1.36) | .11 |

^a^Adjusted for sex, marital status, residence, chronic diseases, smoking status, drinking status, napping time.

^b^RR: relative risk.

^c^Not applicable.

^d^*P*<.0*5*

## Table S4. Participant education by internet use at baseline in the China Health and Retirement Longitudinal Study, 2015

| **Characteristic** | **Overall** | **Internet non-use** | **Internet use** | **P value** |
| --- | --- | --- | --- | --- |
| Education, n (%) | n=15015 | n=14211 | n=804 | <.001a |
| No formal education (illiterate) | 3741 (24.9) | 3734 (26.3) | 7 (0.9) |  |
| Did not finish primary school | 2863 (19.1) | 2846 (20.0) | 17 (2.1) |  |
| Elementary school | 3402 (22.7) | 3330 (23.4) | 72 (9.0) |  |
| Middle school | 3183 (21.2) | 2933 (20.6) | 250 (31.1) |  |
| High school or above | 1826 (12.2) | 1368 (9.6) | 458 (57.0) |  |

^a^*P*<.0*5*

**Table S5. Longitudinal associations between baseline sleep quality and internet use/internet frequency in 2018.**

| **Outcomes** | **Unadjusted Model** | | **Adjusted Model^a^** | |
| --- | --- | --- | --- | --- |
|  | **RR**^b^ **(95%CI)** | **P Value** | **RR (95%CI)** | **P Value** |
| **Sleep quality-Internet use（yes/no）** |  |  |  |  |
| Total Number | 15825 |  | 15361 |  |
| No | 1 (Reference) | N/ A^c^ | 1 (Reference) | N/ A |
| Yes |  |  |  |  |
| good | 1 (Reference) | N/ A | 1(Reference) | N/ A |
| fair | 0.89(0.73,1.08) | .25 | 1.12(0.89,1.42) | .34 |
| poor | 0.55(0.45,0.67) | <0.001^d^ | 0.77(0.62,0.96) | .02^d^ |
| **Sleep quality-Internet frequency** |  |  |  |  |
| Never | 1 (Reference) | N/ A | 1 (Reference) | N/ A |
| Not regularly |  |  |  |  |
| good | 1 (Reference) | NA | 1 (Reference) | N/ A |
| fair | 1.35(0.66,2.74) | .41 | 1.49(0.73,3.04) | .27 |
| poor | 0.44(0.27,0.70) | .001^d^ | 0.56(0.34,0.92) | .02^d^ |
| Almost every week |  |  |  |  |
| good | 1 (Reference) | NA | 1 (Reference) | N/ A |
| fair | 0.63(0.35,1.11) | .11 | 0.68(0.38,1.22) | .20 |
| poor | 0.35(0.18,0.68) | .002^d^ | 0.49(0.25,0.93) | .03^d^ |
| Almost daily |  |  |  |  |
| good | 1 (Reference) | N/ A | 1 (Reference) | N/ A |
| fair | 0.88(0.71,1.08) | .21 | 1.14(0.90,1.45) | .29 |
| poor | 0.58(0.46,0.72) | <0.001^d^ | 0.82(0.65,1.04) | .11 |

RR: relative risks; CI: confidence interval. P < 0.05 employs *italics*.

^a^Adjusted for age, sex, marital status, residence, chronic diseases, smoking status, drinking status, napping time and baseline internet use/internet frequency

^b^RR: relative risk.

^c^Not applicable.

^d^*P*<.0*5*
